# An Overview of the Impact of an Embedded Evaluation of the Immunisation Education, Training and Clinical Support Programme During the New Zealand COVID-19 Pandemic

**DOI:** 10.7759/cureus.101706

**Published:** 2026-01-16

**Authors:** Anthony Dowell, Gayl Humphrey, Maria Stubbe, Susan Bibby, Marama Cole, Abigail Dunlop, Jo Hilder, Larisa Karteleva, Kunal Kumar, Azim O'Shea, Mayor Pokino, Mandy Sexton, Penny Zipfel

**Affiliations:** 1 Primary Health Care and General Practice, University of Otago, Wellington, NZL; 2 The National Institute for Health Innovation, University of Auckland, Auckland, NZL; 3 Primary Health Care and General Practice, Univeristy of Otago, Wellington, NZL; 4 Primary Health Care and General Practice, University of Otago, Wellington , NZL; 5 The Immunisation Advisory Centre, University of Auckland, Auckland, NZL

**Keywords:** appreciative inquiry, complexity and implementation science, covid-19 equity, education and training, embedded evaluation, immunisation

## Abstract

Background

The introduction and roll-out of the COVID-19 vaccine was one of the most important and complex initiatives undertaken by health sectors worldwide. In New Zealand, the Education, Training and Clinical Support to the health sector of the immunisation programme was undertaken by the Immunisation Advisory Centre (IMAC). This paper describes how an embedded evaluation was planned from the outset, acting as a rigorous "critical friend" to the programme: appreciating and recording successes, identifying challenges and opportunities early, and working with stakeholders to generate innovative solutions (Hindsight to Foresight).

Method

The evaluation research team used an established methodology and evidence base, combining complexity and implementation science with appreciative inquiry (CIS-A), previously applied in immunisation strategy and mental health settings. This approach was taken to acknowledge the complexity of the COVID-19 immunisation context.

Both numerical and narrative data were collected to provide information to the programme for endorsement of what was going well and the early identification of challenges. Methods included analysis of IMAC routinely collected workforce data, help line call data, surveys, focus groups and interviews with vaccinators, vaccinees and key stakeholders. There was also direct observation of vaccination sites and settings.

Results

The embedded evaluation process was sustained throughout the COVID-19 immunisation response. Regular assessment and feedback enabled recognition and support of positive features of activity information about challenges with the opportunity for early course correction.

There were specific areas where external evaluation was especially helpful, the focus on important and concise “Toolkit messages for the day” approach and the focus on equity being two examples.

One of the challenges in using an iterative embedded methodology is that what might have been traditional "end of project" evaluation findings have already been raised and, in many cases, acted upon. Changes have therefore already been made and there may be a perception that an evaluation is adding little to project development.

Conclusions

The evaluation was able to assess the overall success and contribution of the programme to the immunisation rollout. We recommend that using a multi-perspective embedded evaluation approach can be of value in implementing complex health sector initiatives.

## Introduction

Background

Following the detection of the first cases of COVID-19 in Wuhan in 2019, health systems undertook varying strategies of containment and response. The introduction and roll-out of COVID-19 vaccines following the first administration in December 2020 was one of the most important and complex initiatives undertaken by health sectors worldwide [1].

Post pandemic, it is important that there is evaluation and learning about the success and challenges encountered in both training an enhanced vaccinator workforce, but also the methods by which successful vaccination strategies can be supported and maintained. This is particularly important given the international decline in immunisation rates in different settings [2,3].

In New Zealand, following the first COVID-19 case on the 23rd of February 2020, the Government introduced an Elimination Strategy of ‘zero-COVID-19’ in March 2020 [4]. This strategy continued until the 3rd of October 2021, providing a rare opportunity in contrast to other countries to plan and prepare a vaccine response without large numbers of COVID-19 patients putting pressure on the health system.

On the 16th of December 2020, the Government announced plans for New Zealand’s largest immunisation roll-out. Prior discussions had begun with the Immunisation Advisory Centre (IMAC) about the development and scope of vaccinator training and clinical support that would be required for such an immunisation roll-out. The IMAC has had a 20-year presence in the country of providing education, training and advice to the health sector in regard to other immunisations and is the only national provider in the country of such programmes.

Prior to COVID, the IMAC had a team of educators and clinicians responsible for training new vaccinators and providing clinical advice to the existing vaccinating workforce and clinicians.

The aim of the initial education programme was "to create a clinically and culturally competent immunisation workforce to deliver COVID-19 vaccines and to upskill the wider health sector to promote immunisations".

The work programme was primarily focused on training healthcare professionals in the public and private sectors. This included the provision of education, training, support and resources to enable primary and secondary care health providers to deliver COVID-19 vaccinations locally.

Out of scope was the development of vaccination education and advice for the members of the public and recruitment of healthcare professionals to complete the COVID-19 vaccinator and COVID-19 Immunisation Register (CIR) training modules.

The greater part of the existing vaccinator workforce consisted of nurses working in primary care and general practice. Doctors were also permitted to give vaccinations, as were pharmacists in some instances.

In New Zealand, the vaccination rollout was mindful of the challenges of inequity in access and uptake of vaccines by different ethnic groups, and a focus in the literature on supporting engagement with Māori (Indigenous people of New Zealand) [5,6] and Pacific communities. There was thus a strong focus on making sure that there was work to develop and implement COVID-19-focused education content and training for the Māori and Pasifika health workforce as required, in order to engage more effectively and safely with Māori and Pasifika communities.

There is relatively little reported research on the preparation of and delivery of vaccinator training programmes for COVID-19.

Problem statement

There is European literature on COVID providing a prompt for vaccination education at the undergraduate level [7] and the CoVE programme from the UK to support promotion of the vaccine [8]. A number of programmes provided training with a focus on discussing vaccine hesitancy [9] and an umbrella review highlighted hesitancy and education and training needs across different health care worker groups [10].

There has also been some focus on competency and assessment, an important issue since COVID-19 vaccines contained different procedures and issues in the immunisation process [11].

Globally, given the scope and scale of the planned rollout, there was also consideration given to education and training methods with the WHO developing online vaccination training packages. Evaluation showed that the online training was well received with measurable impacts on knowledge acquisition [12].

Study aim

Recognising the complex nature of the vaccination programme planned in New Zealand, and the novel nature of many aspects of delivery and implementation, it was felt important to not only include an evaluation, but for that evaluation to support the running of the programme from the outset. Findings were to be integrated iteratively into the development and delivery of the immunisation education, training and clinical support by reporting in real time on progress to the IMAC and the New Zealand Ministry of Health.

This paper describes the development and operation of the evaluation framework over the course of the pandemic response and its use in supporting success in innovation and providing suggestions for "course correction" during that response. 

The overall aim was to assess the impact of the embedded evaluation framework on evaluating the development and delivery of the national COVID-19 vaccinator education, training, and clinical support programme, and to identify factors enabling or constraining effective and equitable workforce capability during the rollout.

Primary evaluation questions

How did the embedded evaluation methodology develop and adapt to the requirements of the immunisation training rollout during the course of the programme?

What was the impact of the embedded evaluation in supporting how effectively the education and training programme supported the development of a clinically competent and culturally safe vaccinator workforce?

How well did the programme adapt to emerging system needs, operational challenges, and equity priorities throughout the rollout?

## Materials and methods

The evaluation used a previously developed methodology and evidence base that had been utilised and published previously in immunisation strategy and mental health research settings [13,14].

The evaluation framework combined well-established methodologies for advancing quality in health services delivery, including the use of mixed quantitative and qualitative research paradigms. 

In this study, the design is a prospective embedded process evaluation, enabling us to explore and share with stakeholders aspects of the infrastructure employed in the various programmes and some examples of successful and challenging outcomes.

The framework was initially developed to stimulate discussion about immunisation strategy at an international level, emphasising the need to acknowledge complexity in relation to immunisation programme development, delivery, and outcome. It was then adapted for use in a youth mental health programme, with successful identification of the use of the approach as an active participant in co-produced positive change.

**Figure 1:**
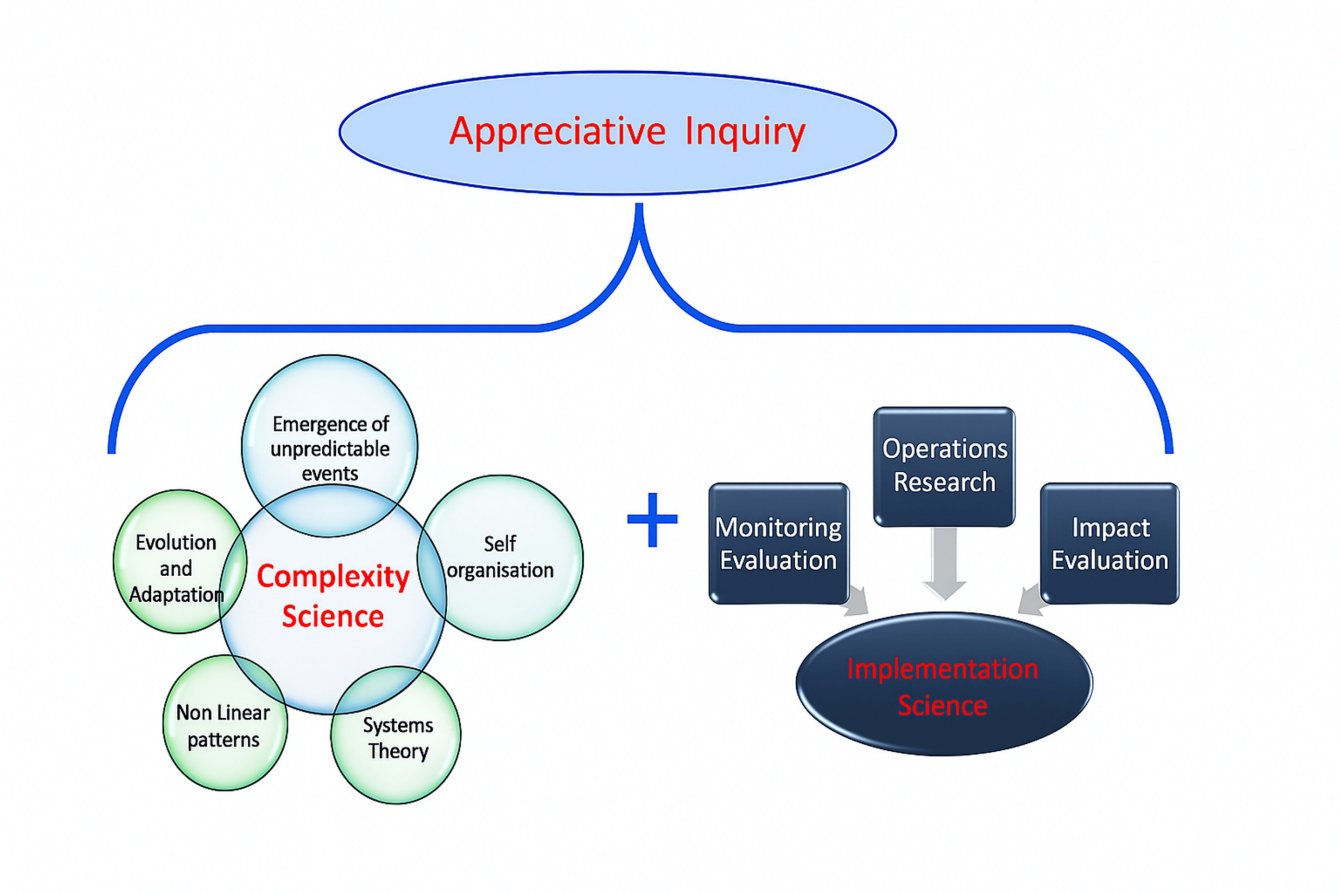
A framework of complexity and implementation science using appreciative inquiry (CIS-A) Image credit: Anthony Dowell

Figure 1 shows the overarching framework used for the evaluation; this is based on the principles of complexity and implementation science, with an underpinning platform of "appreciative inquiry" [15], which seeks to elicit a strengths-based approach to evaluation activity and analysis from the standpoint of a "critical friend". The overall aim of the approach is to support local sustainable innovations and knowledge transfer, built in from the beginning of a programme.

Complexity science approaches acknowledge the need to explore organisational and systems change from multiple perspectives, to utilise non-linear approaches, and to recognise that often ‘predictably unpredictable’ consequences emerge during interventions. It also acknowledges challenges as learning opportunities [16,17]. Implementation Science promotes the systematic uptake of research findings and other evidence into routine practice and also offers insights to enhance the performance of health sector delivery [18].

Using the evaluation framework involved acknowledging the complexity of the immunisation context for COVID-19, and the following core principles outlined below were used in data collection, analysis, and dissemination.

Core principles

The evaluation team sought to appreciate the value of multiple diverse views and perspectives on the strengths and challenges offered within the education, training, and clinical support programmes. Multiple sources of information were brought together to support the identification and resolution of issues and to enable rapid responses to emerging challenges. The evaluation team actively worked with the many different partners, stakeholders, and organisations involved in the training initiative. The evaluation team sought to respond rapidly to emerging issues and provide information about them to the IMAC teams. Local adaptation and context-specific responses to problems were deliberately explored and valued. An overall ‘appreciative inquiry’ approach was taken to understand what was being done within the rapidly changing circumstances of the pandemic. IMAC and the wider health sector brought a wealth of prior practical experience and expertise to the COVID-19 response, and the value of transitioning from existing work platforms was explicitly acknowledged.

Data sources and operational methodology

The mixed methodology undertook a range of projects, collecting both quantitative and qualitative data. Quantitative data was obtained from surveys and exploration of material collected on IMAC databases, and qualitative data from interviews, focus groups and direct observation. A database was created of all vaccinators trained, updated and authorised by type and location. We also collected data on all access made to the Immunisation Website and help line phone calls over time and by theme. Regular collaborative discussions between research teams took place to synthesise different forms of data for feedback to the IMAC and report production. There were regular quarterly reporting meetings with IMAC and additional meetings when significant events occurred or were observed. There were also meetings with other stakeholders to provide information on project progress.

Reviews were undertaken of all the online and in-person courses and webinars delivered by the IMAC. We also reviewed additional produced material such as the COVID-19 toolkit and conducted surveys relevant to the programme. These surveys were to different workforce groupings such as pharmacists and newly developed workforce groupings, as well as health authority stakeholders.

Descriptive statistics were used to describe survey data with summary descriptions of single variables and the associated survey sample. Qualitative data were subjected to initial group consensus review and content analysis so that any "hot topics" and information could be disseminated to the IMAC without delay in the form of "rapid feedback" reports. In addition, extensive and rigorous coding and thematic analysis were undertaken for selected themes at the interim report stage and across all chosen areas of review for this final report. Quarterly reports were submitted to the IMAC throughout the evaluation, with each report containing reflection points and suggestions for review. While the first quarterly and interim reports were appropriately focused on early operational issues pertinent to the first vaccine response, later reports focused in addition on broader strategic themes and evaluation of aspects of the integration of COVID-19 activities with "business as usual" issues.

## Results

How did the embedded evaluation methodology develop and adapt to the requirements of the immunisation training rollout during the course of the programme?

In the course of the evaluation, the team undertook reviews of 12 IMAC online courses, reviewed 28 webinars and other presentations, including versions of a ‘COVID toolkit’, produced and analysed 13 ad hoc surveys and conducted individual and group interviews with 224 individuals in total across 37 organisations (66 internal and external stakeholders),103 service providers across 29 organisations as well as with 55 vaccine recipients (see Appendix 1 for further details). There was also direct observation of pilot training sessions, vaccination processes and vaccination sites throughout the course of the evaluation and reviews of other media and government reporting.

Quarterly reports to the IMAC throughout the evaluation contained reflection points and suggestions for review. While the first reports were appropriately focused on early operational issues pertinent to the initial vaccine response, the interim and later reports focused in addition on broader strategic themes and evaluation of aspects of the integration of COVID-19 activities with 'business as usual’ vaccination issues. In line with the chosen methodology, feedback meetings were held with the IMAC senior leadership team every two weeks, with additional meetings scheduled when additional issues arose.

An initial collection of questions was agreed with the IMAC to form the basis for the evaluation. These questions were framed within Donabedian’s Structure Process and Outcome quality improvement paradigm [19]. During the production of an initial quarterly report, the initial questions were reformatted into higher level evaluation themes and questions to provide a more useful framework for the IMAC and stakeholders to consider current opportunities and challenges.

Overview of outcomes from the education, training and clinical support programme

Data Outputs

The IMAC provided a very significant quantity of educational materials and clinical support to the health sector. Between January 2021 and November 2022, 34,566 unique users/unique logins had completed and passed one or more COVID-19-related courses on the IMAC Learning Management System. Ongoing communication to the workforce was provided from one-on-one clinical advice via the 0800 phone line (around 2,000 per month), to weekly or more regular updates to around 38,000 vaccination and allied workforce subscribers via e-newsletter, around 44,000 users via website clinical advice, and approximately 750 people per month for educational webinars and website information.

New Zealand had traditionally had a relatively standard and single model approach to education, training and clinical support to the vaccination workforce. While this had included a previous quality assurance programme, the IMAC had no previous experience of an embedded iterative evaluation process. The inclusion of the evaluation, which required additional time for reflection and discussion, was at times challenging, particularly during periods of high-pressure activity.

What was the impact of the embedded evaluation in supporting how effectively the education and training programme supported the development of a clinically competent and culturally safe vaccinator workforce?

Examples of Methodology in Action

The evaluation highlighted or introduced a number of suggestions which became part of the IMAC COVID-19 response, and which we present as positive results of the evaluation methodology. They included evaluation observations relating to basic but important practical "on the day" issues which should be highlighted in times of health sector information overload, and also observations relating to longer term strategic issues such as the relationship and boundary between IMAC work and other stakeholders including the Ministry of Health.

We include brief summaries of three examples of evaluation methodology, which we think provided benefit to illustrate the value of the “hindsight to foresight” approach.

Example 1. Direct observation: essentials for safe immunisation: We used CIS-A principles by undertaking multiple and different observational standpoints and being aware of new and emergent events. Whilst the majority of clinical support was very positively received, we noted the potential for information overload (often due to multiple providers being involved) and unavoidable sector ‘busyness’, which led to important information being missed or downplayed. We observed vaccinations being delivered in the wrong anatomical site, and the omission of specific questioning pre- and post-vaccination. We suggested the introduction of a “COVID-19 Toolkit” with concise summaries of what was needed ‘on the day’ to provide an opportunity for further consolidation about the essentials for safe and effective immunisation.

**Figure 2:**
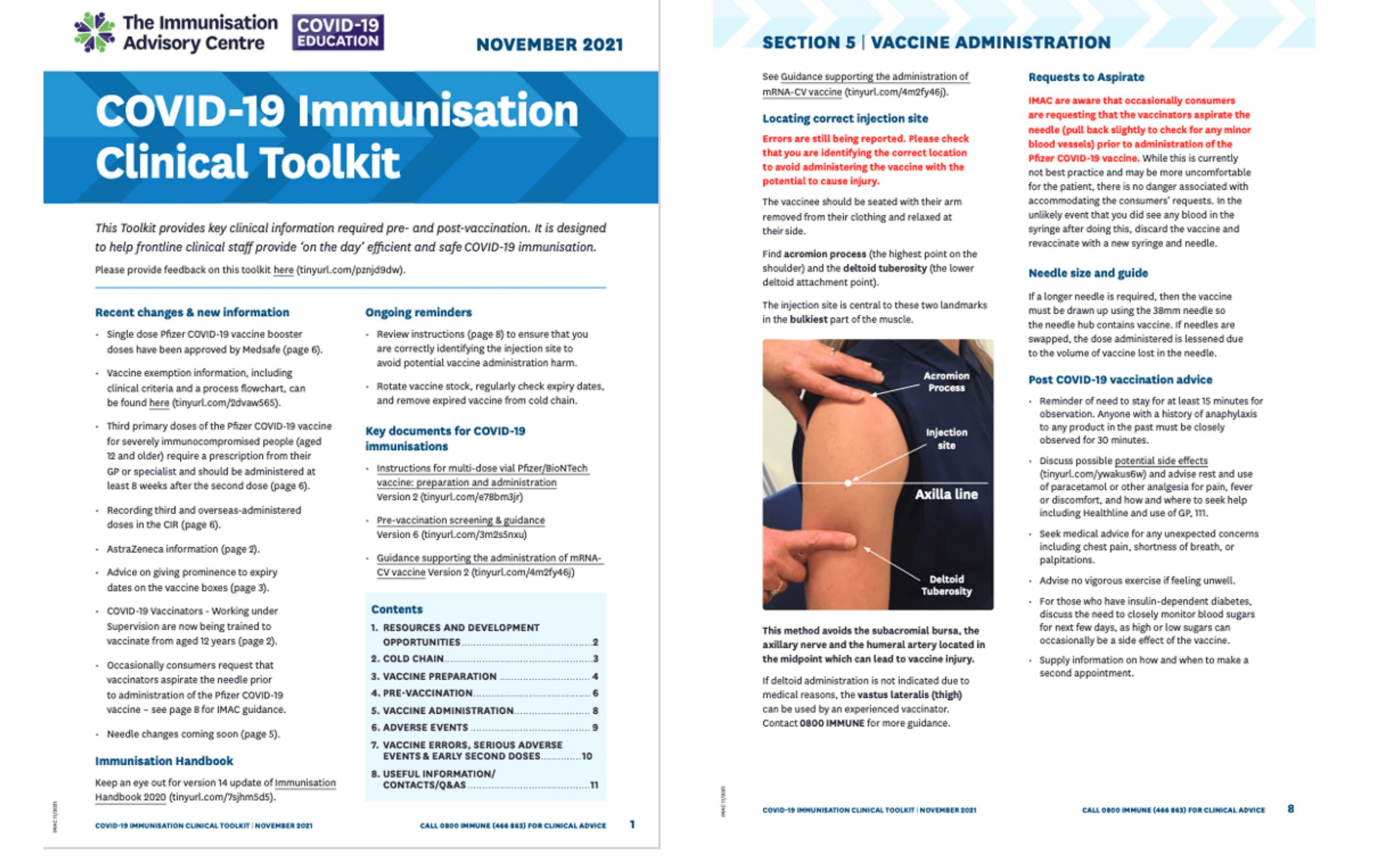
The COVID-19 toolkit Attribution and permission from The Immunisation Advisory Centre [20]

Figure 2 shows the cover and a page insert from a version of the COVID toolkit, with details of issues such as appropriate "draw up" of the vaccine and correct vaccine administration.

Example 2: Support for innovation based on multiple sources of feedback: The CIS-A methodology emphasises the need to obtain multiple perspectives on opportunities and challenges perceived by the evaluation team.

While prior to COVID, immunisation was carried out largely by registered nurses in General Practice, with some support from doctors and pharmacists, the scale of the COVID-19 immunisation rollout required additional workforce development. An important feature of the New Zealand COVID-response was the creation of new innovative workforce roles: the COVID-19 Vaccinator Working Under Supervision (CVWUS), Immunisation Support Worker (ISW), and Vaccinating Health Worker (VHW). Courses for these groups were developed during 2021, along with courses for enhancing the vaccination capability in the pharmacy workforce. IMAC responded well and quickly to the challenge of producing education, training and clinical support for these groups.

The aim of these new workforce initiatives was twofold. Firstly, the CVWUS and ISW were largely to “increase the workforce so that communities have vaccinators who are reflective of their community” and thus improve vaccination rates among Māori and Pasifika. For Pasifika, having vaccinators who speak Pasifika languages was also important. The enhanced pharmacy workforce strategy was aimed at broadening the number of locations that could offer the COVID-19 vaccination.

Feedback gained from the variations between local provider initiatives suggested this was an opportunity to review overall learning paradigms and processes. The evaluation team noted that training people with different backgrounds who were not health professionals brought in people with different strengths and communication styles that could be effective.

Having new vaccinating professionals also meant varied different preferences for learning styles and approaches, for example, the balance between online and face-to-face education and training.

It became clear in the early stages of the pandemic that putting equity as a key driver to all aspects of the immunisation programme would not only be essential to overall success in managing COVID-19 but would be an integral part of supporting communities with known differential to immunisation uptake.

There was external and internal pressure during the initial phases of the COVID-19 response to rapidly adopt a new and different equity-focused approach to work design and outputs, both in how they related to external stakeholders and vaccinators. However, there were challenges in addressing this, especially in the earlier stages of the pandemic response when the health sector was under intense pressure to deliver operationally. We noted and particularly made comments on challenges in rural settings.

The evaluation methodology encouraged incremental innovation in this area, particularly in new vaccinator workforce development, and using immunisation strategies developed within local communities, particularly Māori and Pacific.

Example 3: The views of vaccine recipients: Effective communication with vaccine recipients was seen by vaccinators as a critical part of their role in terms of engaging positively with the recipient, obtaining informed consent, and ensuring the recipient was as relaxed and comfortable as possible throughout the process.

The evaluation team also felt that the views of vaccine recipients were important as they represented several aspects of the end points of education and training. According to CIS-A principles, we felt that vaccine recipient views would be a way to recognise the importance of appreciative inquiry. A series of 12 focus groups (55 participants) and seven individual interviews affirmed an overall positive vaccination experience. The majority of recipients interviewed reported that their vaccinator made them feel welcomed, comfortable and listened to throughout their vaccination procedure.

The vaccinators were actually really good, really chatty, talkative, explained everything with all the technical stuff and then gave you the jab before you knew it you were done and dusted.

(VR11-07, Pasifika, Male, 55-59yr, Auckland)

We were able also to report back important educational reflection points, with a number of vaccine recipients reporting just being ‘jabbed’ without recalling any attempt to landmark the arm, and others feeling rushed, or subject to poor communication skills.

She was like kind of not listening to me, not listening to my thoughts and everything and how my body reacts, and she was just like “No, it must be done this way,”

(VR16-01, NZE, Female, 18-24yr, Auckland)

Varying reports of post-vaccination advice were also obtained from vaccine recipients, an important reflection point the rare, but important adverse reaction reports.

Vaccine recipients also highlighted the need for specific communication skills training for vulnerable groups such as those with physical disability.

In terms of the organisation and setting up of clinics, vaccine recipients clearly indicated that there is no ‘one size fits all’ in terms of the kind of vaccination experience people want. Mass vaccination centres worked really well for those who wanted no fuss and a ‘quick fix’, and staff running these were praised for their organisational skills. By contrast, some vaccinees, particularly Māori, found them a bit cold and clinical and not allowing time for appropriate engagement and communication.

There is clearly much to be learned from vaccinee narratives, and we recommend that this becomes a future component of both ongoing routine and epidemic or pandemic vaccination work.

## Discussion

This paper describes the methodology used to evaluate an immunisation education, training and clinical support response to the COVID-19 pandemic. Throughout the COVID-19 response, the evaluation aimed to be a "critical friend" to the programme, providing suggestions to the provider organisation and attempting communication and liaison with other stakeholders.

Our paper demonstrates the merit in the inclusion of an embedded evaluation process, with the ability to recognise and support positive features of organisational activity and provide information about challenges, with the opportunity for early course correction (Hindsight to Foresight).

The evaluation aligns with the increasing recognition of process evaluations in complex health scenarios [21] and has a focus on mixed methods and multiple data collection sources.

By using an evaluation framework with a focus on appreciative inquiry, we were able to provide positive feedback to IMAC and other stakeholders on multiple occasions. The ability to use appreciative inquiry during evaluation, in this way, we found was helpful using a similar methodology in a mental health setting [14]. Given the stress and pressure generated by COVID-19 and beyond, providing positive feedback is important and often not deliberately included in evaluation system design. 

While there is little direct comparable use of embedded evaluation methodology, several of the important themes emerging from our work, which supported the programme, are recognised in other studies.

The need for robust evaluation of education training was recognised in the RE-AIM framework evaluation in training health workers to be vaccine champions. This measured programme reach, effectiveness, adoption, and implementation using mixed-methods [22].

Our evaluation results highlighted the role and overall success of online training, something recognised globally with the WHO evaluation of open online training enabling rapid confidence in learners' ability to support COVID-19 vaccination following completion of online courses [12].

Our evaluation highlighted the importance of direct observation of education, training, and clinical support activities and resulting in the production of the COVID-19 kit of practical priorities for immunising.

Evaluation of the overall COVID-19 vaccination process in Lebanon occurred through on-site visits to vaccination centres, demonstrating that international standards were generally followed, but some quality deficiencies were identified [23].

In Saudi Arabia, a clinical care and education programme included vaccine handling and storing, procedure for safe administration of COVID-19 vaccine, management of clinical waste, observing adverse events response (AER), management, and reporting, clinical audit, distribution of training materials, and maintenance of training records [24].

Comparative literature has emphasised the role of evaluation support for local incremental innovation. In the UK, the vaccination programme changed from an initial largely centrally controlled strategy into more locally driven and tailored approaches able to respond more effectively to inequalities [25]. This is echoed by additional New Zealand literature. Local Māori initiatives provided additional community activities (e.g., Māori community vaccination days, with provision of rapid Antigen Tests and focus on additional health issues. This was deliberately combined with engagement with "hard to reach" sections of the community and acknowledgement of the role of respected community leaders and elders [26].

As we noted in the innovation seen from Māori providers, other New Zealand research has particularly focused on the rural context where local providers found geographically tailored, culturally anchored, and locally driven solutions [27].

Embedding research and evaluation is increasingly recognised as important in supporting health improvement across a range of different health care settings [14,28,29].

One of the challenges in using an iterative embedded methodology is that what might have been traditional "end of project" evaluation findings have already been raised and acted on; changes have already been made, and there may be a perception that an evaluation is adding little to project development [30].

Limitations

As with much health sector planning and activity during the pandemic, there are limitations to study design, preparedness, and potential follow-up to assess the impact of interventions. New Zealand’s elimination strategy provides a specific context for immunisation training, rollout, and support, which may not be transferable to other international contexts. The research team, along with IMAC and provider organisations, had little opportunity to follow up on the results obtained during the evaluation, given the demands of the ongoing COVID-19 response.

We feel it is important that the learning from COVID-19 immunisation responses is retained in sufficient detail for future pandemic planning. 

## Conclusions

In conclusion, we feel there has been merit in the inclusion of an embedded formative evaluation process, with the ability to recognise and support throughout a programme, positive features of activity, and provide information about challenges with the opportunity for early course correction (Hindsight to Foresight). There are specific areas in this work where we feel early discussion by the evaluation team as ‘critical friend’ has been helpful. The focus on the important and concise COVID-19 toolkit approach and the focus on equity being two examples.

We recommend that using a multi-perspective embedded evaluation approach can be of value in implementing complex health sector initiatives.

## Appendices

Appendix 

**Table 1:** Details of surveys undertaken and qualitative interviewees

No.	Date	Survey Name	Purpose Summary
1	Sep-22	Pharmacy Vaccinator Survey	Experience & role expansion for pharmacists/technicians.
2	Aug-22	CVWUS Authorisation Survey	Reasons for non-completion/delay in CVWUS authorisation.
3	May-22	Bridging Course Feedback	Enablers/barriers for full vaccinator upskilling.
4	May-22	Communication Survey	How vaccinators stay updated with guidelines.
5	Feb-22	DHB Leads Workforce Data Survey	Use of data for planning; key workforce concerns.
6	May-22	ISW Survey	Collated feedback on ISW course.
7	Jan–Feb 2022	Paediatric Pfizer (5–11) Feedback	Module improvement needs.
8	Oct-21	CVWUS Workforce Survey (2nd)	Experience of initial CVWUS cohorts.
9	Oct-21	All COVID Course Completers (3rd)	Training pathway experience; Māori/Pacific sub-analysis.
10	Sep-21	CVWUS Workforce Survey (1st)	Training-to-work pathway; Māori/Pacific sub-analysis.
11	Aug-21	All COVID Course Completers (2nd)	Current work status.
12	May-21	All COVID Course Completers (1st)	Current work status.
13	Mar-21	Health Prescribers Survey	Experience of online training module.
Participant Type	No. of People Interviewed	No. of Organisations	
Stakeholders	66	8	
– IMAC	44	1	
– MoH/Te Whatu Ora	15	2	
– DHB	5	3	
– PHO	1	1	
– College of GPs	1	1	
Service Providers	103	29	
– CVWUS cohort	68	22	
– GP Practices	6	3	
– Iwi/Māori Providers	39	11	
– Pacific-led Providers	12	3	
– Pharmacies	9	4	
– PHO	2	1	
ISW Trainees	8	7	
Other Vaccinators	27	—	
Service Users	55	—	
TOTAL	224	37	
Indicator	Typical Range		
N of responses per course	42 – 7,349		
Most core courses	1,200–3,000		
Newer VHW/Bridging	40–200		
Indicator	Typical Range		
Satisfied with course registration	95–99%		
Logging in straightforward	90–98%		
Indicator	Typical Range		
Modules easy to navigate	94–100%		
Content clear & well organised	97–100%		
Content understandable	93–100%		
Resources supported learning	94–100%		
Indicator	Typical Range		
Knowledge checks helped learning	97–100%		
Confident addressing vaccine concerns	97–100%		
Prepared for practical training	93–99%		
Indicator	Typical Range		
Satisfied with overall quality	96–100%		
Would recommend to colleagues	94–97% (where asked)		
Course Type	Completion Times		
Short top-ups (e.g., VHW)	1.5–3 hours		
Core/full training	3–5 hours		
Adrenaline & AstraZeneca	<3 hours majority		
Competency	Confidence (Typical)		
Face-to-face readiness	95–99%		
Storage & preparation (Pfizer)	96–99%		
Novavax readiness	98–99%		
Infuenza administration	97%		
Anaphylaxis management	97–99%		
Indicator	Result		
Confidence in whānau-centred approach	97–98%		
Theme	Frequency		
Clear content & structure	Very common		
Practical scenarios	Very common		
Knowledge checks reinforcing content	Very common		
Clarity of vaccine information	Very common		
Supportive tone & cultural safety	Common		
Theme	Frequency		
More paediatric EUA updates	Occasional		
Clarifying rare AE pathways	Occasional		
More visuals or flowcharts	Occasional		

Further details of 13 ad hoc surveys, individual and group interviews with 224 individuals and 103 service providers, and 55 vaccine recipients can be found in the background report to this paper (https://ourarchive.otago.ac.nz/esploro/outputs/report/Evaluation-of-IMAC-training-education-and/9926765141501891?institution=64OTAGO_INST).
