# Correction: An Overview of the Impact of an Embedded Evaluation of the Immunisation Education, Training and Clinical Support Programme During the New Zealand COVID-19 Pandemic

**DOI:** 10.7759/cureus.c486

**Published:** 2026-10-06

**Authors:** Anthony Dowell, Gayl Humphrey, Maria Stubbe, Susan Bibby, Marama Cole, Abigail Dunlop, Jo Hilder, Larisa Karteleva, Kunal Kumar, Azim O'Shea, Mayor Pokino, Mandy Sexton, Penny Zipfel

**Affiliations:** 1 Primary Health Care and General Practice, University of Otago, Wellington, NZL; 2 The National Institute for Health Innovation, University of Auckland, Auckland, NZL; 3 Primary Health Care and General Practice, Univeristy of Otago, Wellington, NZL; 4 Primary Health Care and General Practice, University of Otago, Wellington , NZL; 5 The Immunisation Advisory Centre, University of Auckland, Auckland, NZL

The Conflicts of Interest section has been corrected to disclose the following relationships:

- Kunal Kumar and Penny Zipfel declare employment from the Immunisation Advisory Centre (IMAC).
- Anthony Dowell's spouse was Director of the Immunisation Advisory Centre (IMAC) from January to August 2021 and subsequently its Medical Director, during the period covered by this evaluation. IMAC delivered the program evaluated in this article.

